# Synergic impact of oral anticoagulation control and renal function in determining major adverse events in atrial fibrillation patients undergoing percutaneous coronary intervention: insights from the AFCAS registry

**DOI:** 10.1007/s00392-016-1071-0

**Published:** 2017-01-11

**Authors:** Marco Proietti, K. E. Juhani Airaksinen, Andrea Rubboli, Axel Schlitt, Tuomas Kiviniemi, Pasi P. Karjalainen, Gregory Y. H. Lip, On behalf of the AFCAS Study Group

**Affiliations:** 1 University of Birmingham Institute of Cardiovascular Sciences, City Hospital, Dudley Road, Birmingham, B18 7QH UK; 2 Heart Center, Turku University Hospital and University of Turku, Turku, Finland; 3 Division of Cardiology, Laboratory of Interventional Cardiology, Ospedale Maggiore, Bologna, Italy; 4 Department of Medicine III, Martin Luther-University, Halle, Germany; 5 Department of Cardiology, Paracelsus Harz-Clinic, Bad Suderode and Medical Faculty, Martin Luther-University Halle-Wittenberg, Halle, Germany; 6 Heart Center, Satakunta Central Hospital, Pori, Finland; 7 Aalborg Thrombosis Research Unit, Department of Clinical Medicine, Aalborg University, Aalborg, Denmark

**Keywords:** Atrial fibrillation, Percutaneous coronary intervention, Quality of anticoagulation control, Renal impairment, Outcomes

## Abstract

**Background:**

In patients with atrial fibrillation (AF), quality of oral anticoagulation control as well as impaired renal function are associated with adverse outcomes. Our objective was to analyze if there was a synergistic impact of these factors in determining adverse outcomes in AF patients undergoing percutaneous coronary intervention and stent (PCI-S).

**Methods:**

Post-hoc analysis from the Atrial Fibrillation Undergoing Coronary Artery Stenting (AFCAS) registry. Poor oral anticoagulation control was defined as time in therapeutic range (TTR) <65%, while impaired renal function as creatinine clearance (CrCl) <60 ml/min.

**Results:**

Of the whole cohort, 448 were eligible for this post-hoc analysis. Of these, 27.9% had TTR <65%only (Group I), 19.2% had CrCl <60 ml/min only (Group II), while 13.8% had both conditions (Group III). At follow-up, patients in Group III had a higher rate of major adverse cardiovascular and cerebrovascular events (MACCE) (*p* = 0.007), while patients in Groups I and III had higher rates of major bleeding. Kaplan–Meier analyses showed that patients in Group III had higher risk for MACCE (LogRank: 14.406, *p* = 0.003), while Group I and Group III patients had higher risk for major bleeding (LogRank: 12.290, *p* = 0.006). On Cox regression, presence of both conditions independently increased MACCE risk (*p* = 0.001), while TTR <65% alone and the presence of both conditions were independently associated with major bleeding (*p* = 0.004 and *p* = 0.028, respectively).

**Conclusions:**

There was a synergic impact of oral anticoagulation control and renal function in determining major adverse events in AF patients undergoing PCI-S. Use of poor anticoagulation control and impaired renal function in combination would help identify AF patients undergoing PCI-S at risk for MACCE and/or major bleeding.

**Electronic supplementary material:**

The online version of this article (doi:10.1007/s00392-016-1071-0) contains supplementary material, which is available to authorized users.

## Introduction

Atrial fibrillation (AF) is associated with a significant increase in thromboembolic and death risk [1, 2]. Oral anticoagulant (OAC) therapy reduces major adverse coronary and cerebrovascular events (MACCE) in patients with AF [3]. Conversely, treatment with OAC confers a degree of bleeding risk [4].

Despite the introduction of non-vitamin K antagonist oral anticoagulants (NOACs), the vitamin K antagonist (VKA) is still widely used [5, 6]. Quality of OAC control, expressed as time in therapeutic range (TTR), is essential for optimal efficacy and safety [7]. Indeed, low TTR is significantly associated with higher stroke and bleeding rates [8]. Another significant factor influencing morbidity and mortality in AF patients is renal impairment, which itself is associated with higher thromboembolic and bleeding risks compared to those with normal renal function [9]. Of note, renal impairment can significantly influence TTR and its impact on major adverse events [10].

AF patients commonly have associated coronary artery disease, and may undergo percutaneous coronary intervention and stent (PCI-S) [3]. Such patients represent a particularly high-risk group for thromboembolism and bleeding, especially with the necessity to combine OAC with antiplatelet therapy [11]. We, therefore, hypothesized a synergic impact of poor TTR and renal impairment in determining adverse outcomes (MACCE, bleeding) in AF patients. We tested this hypothesis in a post-hoc analysis from the Atrial Fibrillation Undergoing Coronary Artery Stenting (AFCAS) registry.

## Methods

AFCAS was a prospective multicentre European registry including AF patients undergoing PCI-S. Baseline and 1 year outcomes from AFCAS have previously been published [12, 13]. In brief, all patients with on-going or history of AF referred for a PCI-S procedure were eligible to take part in the study. A 12-month follow-up observation period was planned to record all major adverse outcomes. Ethic approval and written informed consent was obtained from every patient, and the study protocol conforms to the 1975 Declaration of Helsinki. For this study, all patients that were prescribed with VKA and had TTR and data about renal function available were considered eligible for this ancillary analysis.

Thromboembolic risk was categorized according to CHA_2_DS_2_-VASc score [14]. “Low-risk” patients were defined as males with a CHA_2_DS_2_-VASc = 0 or females with CHA_2_DS_2_-VASc = 1; “moderate risk” was defined as male patients with CHA_2_DS_2_-VASc = 1; and “high risk” with CHA_2_DS_2_-VASc ≥2.

TTR was calculated according to the Rosendaal interpolation method [15]. INR considered for TTR calculation were performed at baseline and, subsequently at every follow-up visits (1, 3, 6, 12 months), when performed on-site. Poor anticoagulation control was defined as TTR <65%, consistent with previous studies [16, 17]. Renal function was evaluated according to CrCl calculated with the four-item Cockroft–Gault formula [(140−age) × (weight in kg) × (0.85 if female)/(72 × creatinine)]. Accordingly, an estimated CrCl level of <60 ml/min was used to define impaired renal function, again based on established guidelines [18].

Based on the original protocol, the principal efficacy outcome was a composite of MACCE, including acute MI, target vessel revascularization, stroke/transient ischemic attack (TIA), systemic embolic event, stent thrombosis and cardiovascular death. Acute MI was defined according to the universal definition in use at the time of the study [19]. Target vessel revascularization was defined as PCI-S or coronary bypass surgery in the previously treated vessel. Stent thrombosis was defined according to the Academic Research Consortium classification and included definite and probable events [20]. TIA was defined as a focal, transient (<24 h) neurological deficit adjudicated by a neurologist, whereas stroke was defined as a permanent, focal, neurological deficit adjudicated by a neurologist and confirmed by computed tomography/magnetic resonance imaging. Systemic embolism was defined as signs/symptoms of peripheral ischemia associated or not with a positive imaging test. Cardiovascular death was defined as a death related to cardiac cause or stroke. The principal safety outcomes were ‘major bleeding’, defined as intracranial bleeding, bleeding requiring blood transfusion or surgical/endoscopic treatment or leading to long-term disability or death.

### Statistical analysis

Continuous variables were reported as median [IQR] and differences between subgroups were assessed with Kruskal–Wallis one-way ANOVA test with pairwise comparisons between groups. Categorical variables, expressed as counts and percentages, were analysed by Chi-squared test.

Differences in survival between groups were examined with log-rank test and Kaplan–Meier curves were drafted accordingly. Cox regression analysis, adjusted for age, sex, type of AF, CHA_2_DS_2_-VASc score and antithrombotic therapy pattern at discharge, was performed to establish the relationship between TTR, CrCl and their synergic effect in determining MACCE during follow-up observation. A two-sided *p* value <0.05 was considered statistically significant. The sensitivity and specificity of using TTR <65% and CrCl <60 ml/min alone or the combination of both was performed, to identify those AF patients undergoing PCI-S who are more likely to have a major adverse event during follow-up. All analyses were performed using SPSS v. 22.0 (IBM, NY, USA).

## Results

Among 963 patients enrolled in the study original cohort, 448 (46.5%) were eligible for this post-hoc ancillary analysis. Of these 125 (27.9%) had a TTR <65% only (Group I), 86 (19.2%) had a CrCl <60 ml/min only (Group II), while 62 (13.8%) had both conditions, i.e., TTR <65% andCrCl <60 ml/min (Group III). Conversely, 175 (39.1%) patients had none of the two criteria (Group IV).

Baseline conditions are shown in Table 1. Patients in Groups II and III were older (*p* < 0.001) and more likely female (*p* < 0.001), and had lower BMI compared to those in Group I and IV (*p* < 0.001). Except for proportion of patients with diabetes mellitus, that was higher in Group I (*p* = 0.025), baseline characteristics were similar across the four groups. Given the difference in age and proportion of female patients, the mean CHA_2_DS_2_-VASc score was higher in Groups II and III (*p* < 0.001), as was the proportion with high thromboembolic risk (CHA_2_DS_2_-VASc ≥2) (*p* < 0.001). No significant differences were found in the prescription (*p* = 0.867) or duration (*p* = 0.215) of antithrombotic therapy across the four groups (Table 1).

**Table 1:** Baseline characteristics of study cohort

	Group I TTR <65%, *n* = 125	Group II CrCl <60 ml/min, *n* = 86	Group III Both conditions, *n* = 62	Group IV Neither TTR < 65% or CrCl < 60 ml/min, *n* = 175	*p*
Age, (years) median [IQR]	72 [66–75]^a^	79 [74–83]^b^	78 [74–80]^b^	71 [63–77]^a^	<0.001
Female, *n* (%)	29 (23.2)	38 (44.2)	23 (37.1)	35 (20.0)	<0.001
BMI, (kg/m^2^) median [IQR] 433	29 [26–32]^a^	25 [23–28]^b^	26 [24–29]^b^	29 [26–31]^a^	<0.001
CrCl, (ml/min) median [IQR]	80.9 [69.6–109.0]^a^	48.5 [39.9–55.0]^b^	46.3 [35.1–51.7]^b^	84.3 [70.8–106.3]^a^	<0.001
TTR, (%) median [IQR]	37.4 [16.1–50.0]^b^	92.7 [81.0–100]^a^	37.7 [8.2–53.7]^b^	98.9 [86.2–100]^a^	<0.001
AF Type, *n* (%) 444					0.531
Paroxysmal	39 (31.7)	29 (34.1)	16 (25.8)	42 (24.1)	
Persistent	13 (10.6)	5 (5.9)	7 (11.3)	18 (10.3)	
Permanent	71 (57.7)	51 (60.0)	39 (62.9)	114 (65.5)	
Hypertension, *n* (%)	101 (80.8)	71 (82.6)	52 (83.9)	132 (75.4)	0.373
Hypercholesterolemia, *n* (%)	81 (64.8)	53 (61.6)	40 (64.5)	120 (68.6)	0.716
Diabetes mellitus, *n* (%)	52 (41.6)	24 (27.9)	18 (29.0)	45 (25.7)	0.025
Smoking habit, *n* (%)	14 (11.2)	4 (4.7)	4 (6.5)	15 (8.6)	0.363
Previous coronary artery disease, *n* (%)	45 (36.0)	31 (36.0)	23 (37.1)	56 (32.0)	0.830
Previous MI, *n* (%)	35 (28.0)	24 (27.9)	17 (27.4)	35 (20.0)	0.318
Previous PCI, *n* (%)	19 (15.2)	10 (11.6)	7 (11.3)	23 (13.1)	0.844
Previous CABG, *n* (%)	21 (16.8)	12 (14.0)	15 (24.2)	28 (16.0)	0.396
Chronic heart failure, *n* (%)	26 (20.8)	19 (22.1)	13 (21.0)	26 (14.9)	0.405
Ejection fraction, (%) median [IQR] 323	50 [40–60]	50 [40–58]	50 [40–60]	51 [40–60]	0.507
Previous stroke/TIA, *n* (%)	20 (16.0)	19 (22.1)	13 (21.0)	36 (20.6)	0.672
Previous bleeding, *n* (%)	5 (4.0)	4 (4.7)	1 (1.6)	10 (5.7)	0.595
CHA_2_DS_2_-VASc, median [IQR]	3 [2–5]^a^	4 [3–5]^b^	4 [3–5]^b^	3 [2–4]^a^	<0.001
Thromboembolic risk, *n* (%)					<0.001
Low risk	0 (0)	0 (0)	0 (0)	5 (2.9)	
Moderate risk	11 (8.8)	0 (0)	0 (0)	24 (13.7)	
High risk	114 (91.2)	86 (100.0)	62 (100.0)	146 (83.4)	
PCI clinical indication, *n* (%)					
Stable angina	59 (47.2)	31 (36.0)	25 (41.0)	91 (52.0)	
NSTE-ACS	43 (34.4)	34 (39.5)	27 (44.3)	62 (35.4)	
STEMI	15 (12.0)	17 (19.8)	8 (13.1)	14 (8.0)	0.137
Other	8 (6.4)	4 (4.7)	1 (1.6)	8 (4.6)	
PCI clinical setting, *n* (%)					0.158
Emergency	63 (50.4)	34 (39.5)	29 (46.8)	99 (56.6)	
Urgency	44 (35.2)	38 (44.2)	25 (40.3)	62 (35.4)	
Elective	18 (14.4)	14 (16.3)	8 (12.9)	14 (8.0)	
N^o^ diseased vessels, median [IQR] 447	2 [1–3]	2 [1–3]	2 [1–3]	2 [1–3]	0.684
N^o^ treated vessels, median [IQR]	1 [1–1]^a^	1 [1–1]^a^	1 [1–1]^a^	1 [1–1]^a^	0.049
Lesion severity, *n* (%) 414					0.080
A	23 (20.9)	8 (10.3)	8 (14.8)	24 (14.0)	
B1/B2	68 (61.8)	59 (75.6)	32 (59.3)	103 (59.9)	
C	19 (17.3)	11 (14.1)	14 (25.9)	45 (26.2)	
Complete revascularization, *n* (%) 440	60 (49.6)	35 (40.7)	24 (40.7)	89 (51.1)	0.280
Stent type, *n* (%) 434					0.196
DES	28 (23.7)	14 (16.9)	19 (30.6)^#^	44 (25.7)	
Bioactive	16 (13.6)	15 (18.1)	10 (16.1)^#^	42 (24.6)	
BMS	70 (59.3)	49 (59.0)	31 (50.0)^#^	78 (45.6)	
Other	4 (3.4)	5 (6.0)	2 (3.2)^#^	7 (4.1)	
Prescribed antithrombotic therapy, *n* (%)					0.867
Single AP + VKA	14 (11.2)	9 (10.5)	7 (11.3)	15 (8.6)	
Dual AP + VKA	111 (88.8)	77 (89.5)	55 (88.7)	160 (91.4)	
Antithrombotic therapy duration, *n* (%)					0.215
0–3 months	66 (52.8)	50 (58.1)^#^	30 (48.4)	83 (47.4)^#^	
3–6 months	21 (16.8)	21 (24.4)^#^	11 (17.7)	41 (23.4)^#^	
≥6 months	38 (30.4)	15 (17.4)^#^	21 (33.9)	51 (29.1)^#^	

^a, b^ Differences in superscripts between the groups is related to significant differences in pairwise comparisons between groups

*ACS* acute coronary syndrome, *AF* atrial fibrillation, *BMS* bare metal stent, *CABG* coronary artery by-pass graft, *CrCl* creatinine clearance, *DES* drug eluting stent, *IQR* interquartile range, *MI* myocardial infarction, *NSTE* non ST elevation, *PCI* percutaneous coronary intervention, *STEMI* ST elevation myocardial infarction, *TIA* transient ischemic attack, *TTR* time in therapeutic range

^#^Total percentage is equal to 99.9% due to rounded figures

### Follow-up

**Fig. 1:**
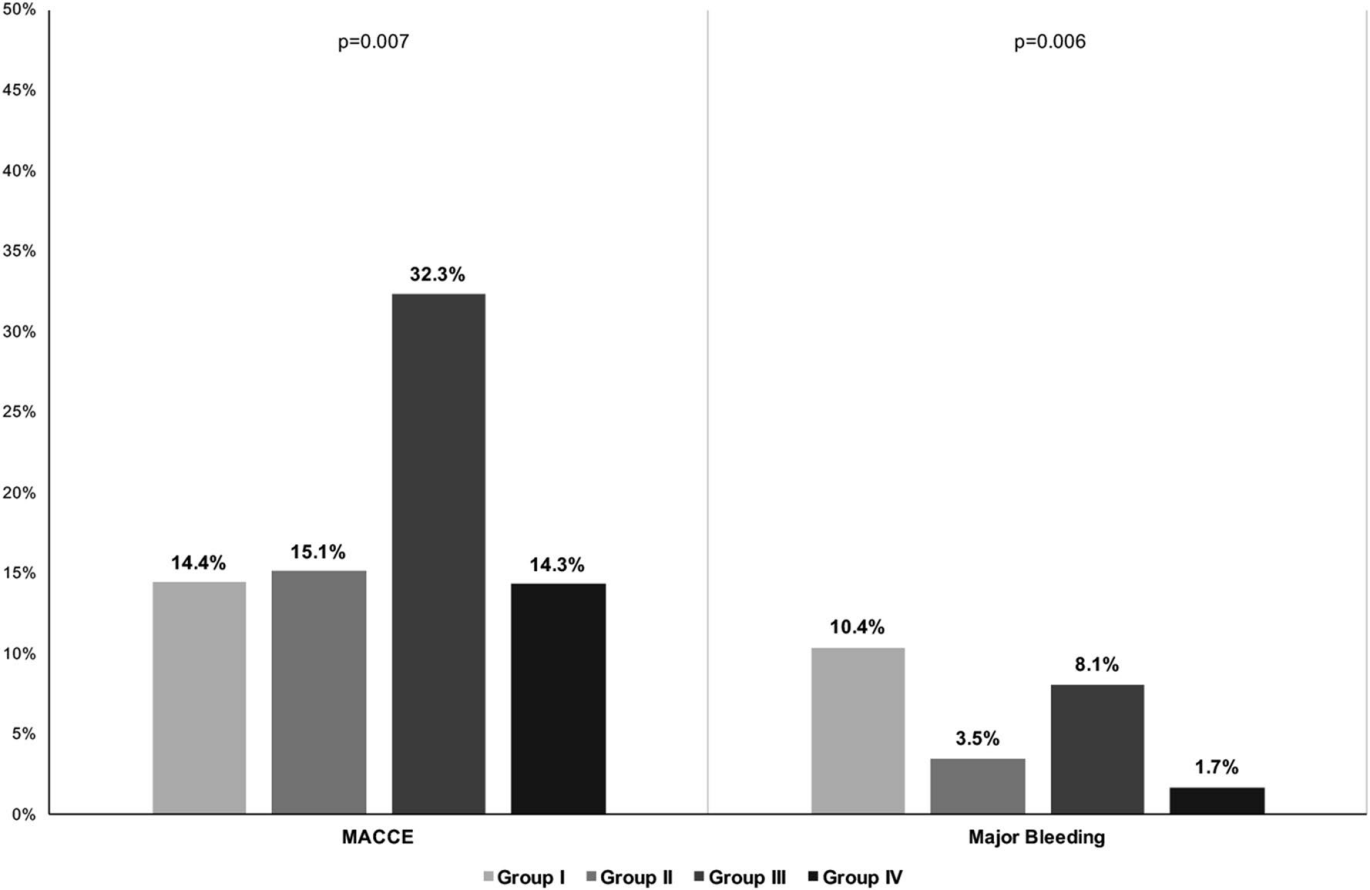
Major adverse events rates according to presence of low-quality anticoagulation and moderate renal impairment. *CrCl* creatinine clearance, *MACCE* major adverse coronary and cerebrovascular events, *TTR* time in therapeutic range

After a median [IQR] follow-up time of 359 [342–369] days, a total of 76 (17.0%) MACCE and 24 (5.4%) major bleeding events were recorded, with an overall incidence of 18.7 per 100 patient-years and 5.7 per 100 patient-years, respectively. Comparing the four subgroups, Group III had a higher MACCE rate compared to other groups (*p* = 0.007) [Fig. 1, Left Side]. For major bleeding events, Groups I and III had higher event rates compared to other groups (*p* = 0.006) [Fig. 1, right side]. Details about individual MACCE are reported in Table S1. A significant higher rate of cardiovascular death was found in Group III (*p* < 0.001) compared to other groups.

**Fig. 2:**
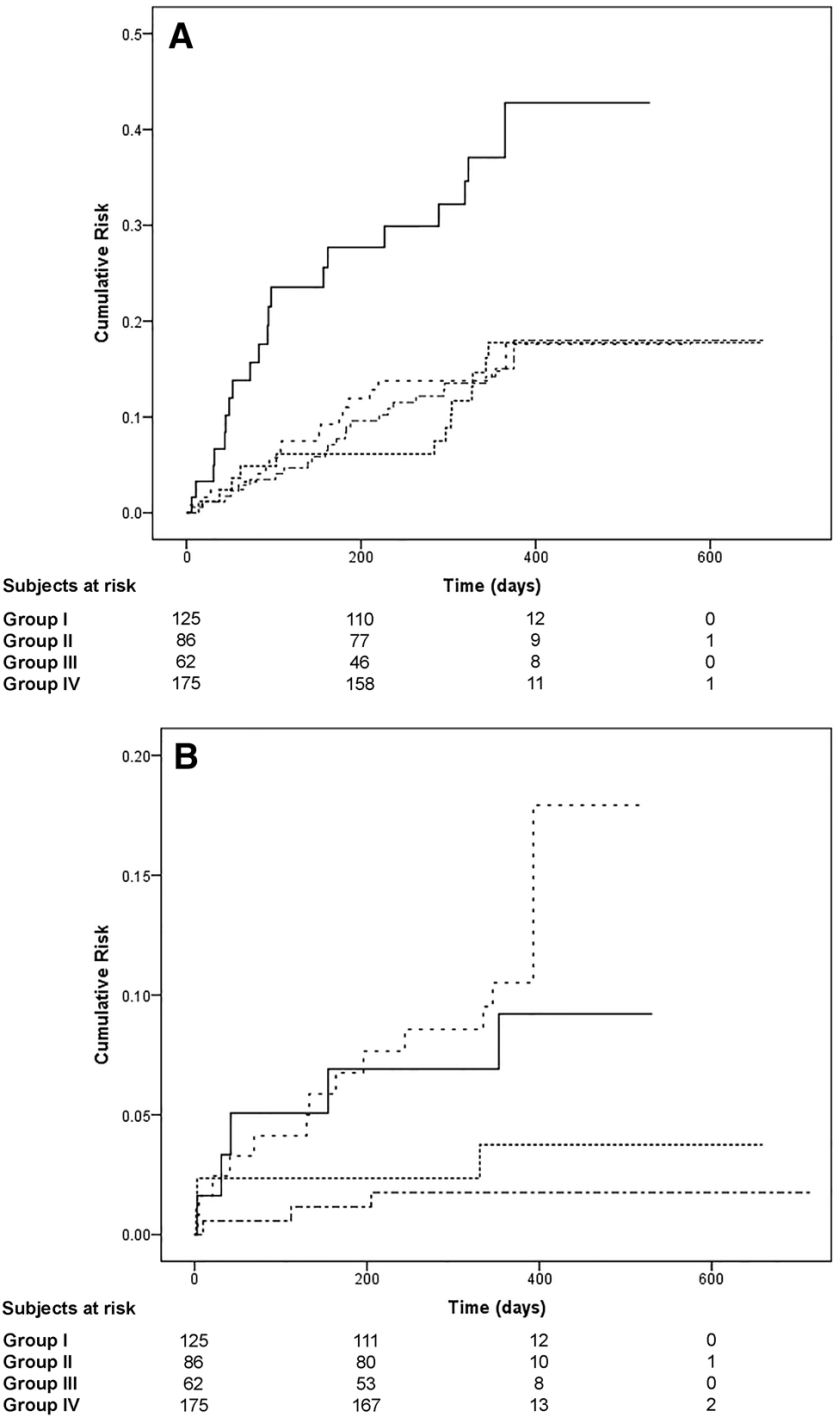
Kaplan–Meier curves for major adverse events. Panel (**a**) MACCE; panel (**b**) major bleeding. *Large dashes* Group I, *Narrow dashes* Group II, *Solid line* Group III, *Alternate dashes* Group IV, *CrCl* creatinine clearance, *MACCE* major adverse coronary and cerebrovascular events, *TTR* time in therapeutic range

Kaplan–Meier analyses showed that when both conditions were present (Group III) there was a higher risk for MACCE (og rank: 14.046, *p* = 0.003) compared to other groups [Fig. 2, Panel a]. TTR <65% alone (Group I) and both conditions together (Group III) had higher risk for major bleeding (log rank: 12.290, *p* = 0.006) [Fig. 2, Panel b].

On Cox regression analysis (Table 2), the presence of both conditions (i.e., TTR <65% and CrCl <60 ml/min) independently increased MACCE risk (*p* = 0.001), while TTR <65% and the presence of both conditions (i.e., TTR <65% and CrCl <60 ml/min) were independently associated with major bleeding (*p* = 0.004 and *p* = 0.028, respectively).

**Table 2:** Cox regression analysis for major adverse events

	HR	95% CI	*p*
MACCE			
TTR <65%	1.04	0.57–1.91	0.896
CrCl <60 ml/min	1.10	0.56–2.16	0.776
Both conditions	2.62	1.46–4.72	0.001
None condition (reference)	–	–	–
Major bleeding event			
TTR <65%	6.24	1.83–22.58	0.004
CrCl <60 ml/min	2.26	0.45–11.26	0.318
Both conditions	4.96	1.18–20.78	0.028
None condition (reference)	–	–	–

Adjusted for age, sex, type of AF, CHA_2_DS_2_-VASc score, antithrombotic therapy

*CI* confidence interval, *CrCl* creatinine clearance, *HR* hazard ratio, *TTR* time in therapeutic range

### Sensitivity and specificity

For MACCE occurrence, TTR <65% had a sensitivity of 50.0% (95% CI: 38.3–61.7%) and specificity of 60.0% (95% CI: 54.8–65.0%). CrCl <60 ml/min had a sensitivity of 43.4% (95% CI: 32.1–55.3%) and specificity of 69.1% (95% CI: 64.1–73.8%). The combination had a higher specificity (88.7%, 95% CI: 85.1–91.7%), but lower sensitivity (26.3%, 95% CI: 16.9–37.7%).

For major bleeding occurrence, the combination had low sensitivity (20.8%, 95% CI: 7.1–42.2%) but high specificity (86.6%, 95% CI: 82.9–89.7%), compared to TTR <65% only (sensitivity 75.0%, 95% CI: 53.3–90.2%; specificity 60.1%, 95% CI: 55.3–64.8%) or CrCl <60 ml/min only (sensitivity 33.3%, 95% CI: 15.6–55.3%; specificity 67.0%, 95% CI: 62.3–71.4%).

## Discussion

In this post-hoc ancillary analysis from the AFCAS study, our main finding was that the concomitant presence of both poor OAC control (TTR <65%) and impaired renal function (CrCl <60 ml/min) independently increased the occurrence of MACCE. For major bleeding, the presence of both conditions was again an independent risk factor, while poor OAC control per se was an independent risk factor for major bleeding, but not renal impairment. Using poor anticoagulation control and impaired renal function in combination would help identify AF patients undergoing PCI-S who are at risk of having a MACCE and/or a major bleeding event during follow-up.

The relationship between renal impairment and adverse events in AF patients has been well-established [9]. Several pathophysiological mechanisms have been proposed in determining the higher thromboembolic and bleeding risks in AF patients with chronic kidney disease (CKD) [9]. More recent evidence shows how progressive worsening of renal function is associated with changes in fibrin clot structure, leading to a progressive higher clot density [21]. Unsurprisingly, epidemiological and clinical trial data clearly demonstrate that in AF patients, CKD increases the risk of stroke [10, 22–26]. Indeed, “impaired renal function” was proposed to increase the predictive ability of stroke risk prediction scoring schemes in a highly selected anticoagulated trial cohort [27]. Despite a modest statistical improvement in predictive ability of clinical scores in the original derivation cohort, this approach was not validated in large “real-world” independent cohorts of AF patients [28, 29].

A recent meta-analysis provides evidence that warfarin treatment reduces the risk of thromboembolic events in AF patients with CKD [hazard ratio (HR): 0.39, 95% confidence interval (CI): 0.18–0.86, *p* < 0.00001] [30]. Indeed, Bonde et al. report a significant net clinical benefit for VKA amogst CKD patients at a high risk of thromboembolic events (CHA_2_DS_2_-VASc ≥2) [25]. Paradoxically, a higher bleeding risk has also been reported in AF CKD patients [31, 32].

VKA treatment is effective and safe when there is good quality OAC control [7]. A European Society of Cardiology Anticoagulation Task Force consensus statement recommends that good anticoagulation control is defined as a TTR >70% [33]. Indeed, lower TTR values are associated with higher risks of thromboembolic and bleeding events [8, 17, 34, 35].

A relationship between quality of OAC control and renal impairment has been reported. For example, an analysis coming from the Outcomes Registry for Better Informed Treatment of Atrial Fibrillation (ORBIT-AF) registry found that the presence of CKD was significantly associated with a low TTR [36]. In a post-hoc analysis from the Stroke Prevention using an Oral Thrombin Inhibitor in patients with atrial Fibrillation (SPORTIF) III and V trials, there was a significant linear relationship between CrCl (expressed with Cockroft–Gault equation) and TTR [10]. Furthermore, the presence of CKD could modify the relationship between TTR and risk for both stroke and major bleeding, conferring a higher risk even with the same level of anticoagulation control [10].

The role of renal impairment in determining adverse outcomes amongst patients undergoing PCI-S has been demonstrated in general populations, both in terms of major cardiovascular events [37] and major bleeding [38]. Similar data have been already described in patients with AF receiving PCI-S [39]. The present study results, in the context of the available evidence, reinforces the concept that quality of OAC control and renal impairment are strongly interconnected in determining the occurrence of major adverse outcomes in AF patients undergoing PCI-S. Our study also reinforces the importance of good quality OAC control in determining major bleeding amongst patients with renal impairment, at least in this specific clinical scenario.

### Limitations

Main limitation to this study is its post-hoc observational nature. The relatively small numbers of patients and the lack of NOAC data could limit the generalizability of the results to contemporary clinical practice.

In conclusion, there was a synergic impact of OAC control and renal function in determining major adverse events (MACCE and major bleeding) in AF patients undergoing PCI-S. Use of poor anticoagulation control and impaired renal function in combination would help identify AF patients undergoing PCI-S who are at risk of MACCE and/or major bleeding.

## Electronic supplementary material

Below is the link to the electronic supplementary material.

Supplementary material 1 (DOCX 14 KB)
