# Supplementary material for: Synergic impact of oral anticoagulation control and renal function in determining major adverse events in atrial fibrillation patients undergoing percutaneous coronary intervention: insights from the AFCAS registry
Source: Clin Res Cardiol. 2017 Jan 11;106(6):420–7. doi: 10.1007/s00392-016-1071-0 (PMC5442242; doi:10.1007/s00392-016-1071-0)
Supplement: Supplementary file 1 — Supplementary material 1 (DOCX 14 KB) [file 392_2016_1071_MOESM1_ESM.docx]

**Table S1:** Occurrence of Individual MACCE Events at Follow-Up

|  | **Group I**  **TTR<65%**  n= 125 | **Group II**  **CrCl<60 ml/min**  n= 86 | **Group III**  **Both Conditions**  n= 62 | **Group IV**  **Neither TTR <65% or CrCl<60 ml/min**  n= 175 | **p** |
| --- | --- | --- | --- | --- | --- |
| **Stroke/TIA**, n (%) | 2 (1.6) | 3 (3.5) | 0 | 5 (2.9) | 0.461 |
| **SEE**, n (%) | 0 | 1 (1.2) | 1 (1.6) | 1 (0.6) | 0.570 |
| **Stent Thrombosis**, n (%) | 1 (0.8) | 1 (1.2) | 0 | 2 (1.1) | 0.858 |
| **Target Vessel Revascularization**, n (%) | 11 (8.8) | 4 (4.7) | 9 (14.5) | 14 (8.0) | 0.203 |
| **AMI**, n (%) | 5 (4.0) | 5 (5.8) | 6 (9.7) | 5 (2.9) | 0.162 |
| **CV Death**, n (%) | 5 (4.0) | 4 (4.7) | 11 (17.7) | 6 (3.4) | <0.001 |

**Legend:** AMI= acute myocardial infarction; CV= cardiovascular; SEE= systemic embolic event; TIA= transient ischemic attack.
